# Latent classes of men’s intimate partner violence perpetration and attitudes towards gender norms: A UN multi-country, cross-sectional study in Asia and the Pacific

**DOI:** 10.1371/journal.pone.0264156

**Published:** 2022-09-26

**Authors:** Tiara C. Willie, Marina Katague, Nafisa Halim, Jhumka Gupta

**Affiliations:** 1 Department of Mental Health, Johns Hopkins Bloomberg School of Public Health, Baltimore, MD, United States of America; 2 Department of Social and Behavioral Sciences, Yale School of Public Health, New Haven, CT, United States of America; 3 Department of Global Health, Boston University, Boston, MA, United States of America; 4 Department of Global and Community Health, George Mason University, Fairfax, VA, United States of America; Washington University in St. Louis, UNITED STATES

## Abstract

**Objective:**

To examine distinct patterns of IPV perpetration and examined gender equitable attitudes as a correlate of these patterns among men from six countries in Asia and the Pacific.

**Design:**

2011–12 UN Multi-country Study on Men and Violence cross-sectional study.

**Setting:**

Households in Bangladesh, Cambodia, China, Indonesia, Sri Lanka and Papua New Guinea.

**Participants:**

10,178 men aged 18–49 years residing in Bangladesh, Cambodia, China, Indonesia, Sri Lanka and Papua New Guinea.

**Primary outcomes measure:**

Our primary outcome was distinct patterns of IPV perpetration which were derived from multilevel latent class analyses.

**Results:**

The odds of being assigned to the Low All Forms of IPV Perpetration class than the High All Forms of IPV Perpetration class was lower for men in the middle tertile group than men in the high tertile group for gender equitable attitudes. The odds of being assigned to the High Emotional IPV Perpetration class than the High All Forms of IPV Perpetration class was greater for men in the low tertile group than men in the high tertile group for gender equitable attitudes. The odds of being assigned to the High Physical/Emotional/Economic IPV Perpetration class than the High All Forms of IPV Perpetration class was lower for men in the low tertile group than men in the high tertile group for gender equitable attitudes.

**Conclusions:**

Gender transformative interventions that use an adaptive, personalized approach to men’s typology of IPV perpetration may be beneficial to reduce violence against for women in the Asia-Pacific region.

## Introduction

Multisectoral efforts are needed to respond to intimate partner violence (IPV) as a human rights violation and serious threat to global public health [1], as women are more at risk of experiencing violence in intimate relationships than experiencing violence in any other context [2]. One in three women are estimated to experience IPV over their lifetime [3]. Several social, structural, and cultural factors contribute to the overwhelming burden of IPV include gender norms and inequities [4, 5]. Intimate partners who perpetrate physical or sexual violence also tend to practice controlling behaviors [2], which all serve to threaten the health, social well-being, and economic growth of victims. IPV can result in adverse health outcomes [6], including mental health [7], physical health [8], and sexual health [9, 10].

Prevention research examining the different forms of IPV perpetration among men in Asia and the Pacific is further warranted. Southeast Asia has a high prevalence (37.7%) of physical and sexual IPV among ever-partnered women worldwide [3]. In the countries included in the current study, gender-based violence behaviors, such as domestic violence and spousal abuse, are especially pervasive [5, 11]. For example, 75% of married Bangladeshi women in a nationally representative sample experienced physical and/or sexual violence [12]. According to the UN Multi-country Study on Men and Violence, a cross-sectional, multicountry population-based study in the Asia-Pacific Region, the prevalence of IPV varies with the highest estimates reported in Papua New Guinea [5].

Despite the pervasive prevalence of IPV across the Asia-Pacific region, research seeking to understand male perpetration of different IPV behaviors in this region has recently emerged. More research directly recruiting men is needed to understand the underlying drivers of violent behaviors to transform the sociocultural context which makes violence by men against women a societal norm. Although factors associated with men’s perpetration of violence against women varied across low- and middle-income countries, IPV was found to be related to unequal gender norms and power inequalities in Bangladesh [1] and Cambodia [5], the Democratic Republic of Congo [13], and Uganda [14]. Indeed, despite a majority of respondents reporting they believed in gender equality across the included sites in Asia and the Pacific, rape was most commonly motivated by a sense of entitlement [14]. Evidence suggests that men’s attitudes about gender norms, and their perpetuation through the social reproduction of these norms in institutions and culture, directly influence behavior [15], including the perpetration of physical and sexual violence [16].

Further, violence is a form of discipline used to enforce gender norms [17, 18]. Gender-transformative approaches that engage men’s attitudes and behaviors have shown to be a promising approach to reduce IPV [19]. Notably, existing intervention studies have had differential impacts on various forms of IPV [2–5]. Investigating differences in men’s attitudes and how they relate to patterns of IPV perpetration may help inform how and for whom these approaches may be the most efficacious.

Identifying subgroups of men who perpetrate IPV to identify differing attitudes toward gender norms may help tailor intervention for violence prevention. Latent class analysis (LCA) is a person-centered approach used to identify subgroups based on patterns of behavior [21, 22], which has recently been used to understand heterogeneity in IPV experiences globally [23–26]. LCA may advance our understanding of IPV perpetration by investigating diversity of relationships between violence-related variables [27], as specific gender equitable norms may be driving specific patterns of IPV against women. Teasing apart underlying factors by behaviors may serve to inform nuanced approaches for programming in the future. To date, the authors are unaware of any study that has adopted this approach to examine IPV perpetration in the region of Asia and the Pacific or any other LMIC.

The current study uses data from the UN Multi-country Study on Men and Violence in Asia and the Pacific to conduct the following aims: (1) use LCA to identify distinct typologies of men based on their perpetration of intimate partner violence, and (2) examine gender equitable attitudes as correlates of men’s IPV perpetration typologies.

## Materials and methods

The methods below have been described elsewhere [6–8].

### Study procedures

The current analytic sample data were drawn from the UN Multi-country Study on Men and Violence, a cross-sectional, multi-country population-based quantitative household survey of 10,178 men and 3,106 women in nine sites across six countries (Bangladesh, Cambodia, China, Indonesia, Sri Lanka and Papua New Guinea). The study was a collaboration between the Partners for Prevention with the Medical Research Council of South Africa and country research teams. The survey was administered from 2011 to 2012. In every research site, a representative sample of men aged 18–49 were recruited using a multi-stage cluster sample strategy. Male participants were interviewed by male interviewers and data was collected using personal digital assistants. All research sites had a response rate of 70%, except in Sri Lanka, which had a response rate of 59%. The study followed international ethical and safety standards for research on violence against women [9]. In the original study, participants were asked to sign consent forms if they felt comfortable or give verbal consent to participate.

The present study was exempted by George Mason University IRB Number: 1503280–1 because the analyses were secondary, and data were de-identified. Given our focus on men’s perpetration of IPV, we restricted our analytic sample to male participants across all research sites (N = 10,178).

### Patient and public involvement

Since the current study is a secondary data analysis of deidentified participant data, it was not appropriate or possible to involve patients or the public in the design, or conduct, or reporting, or dissemination plans of our research study.

### Measures

Latent classes were based on 18 behaviors related to lifetime. Men were asked a series of behavior-specific questions related to current or former IPV based on the WHO Multi-country Study on Women’s Health and Domestic Violence questionnaire for women [10], and a South African survey adapted for men [11]. Men were asked questions related to perpetration of five acts of emotional abuse (e.g., belittling, insults), five acts of economic abuse (e.g., prohibited her employment), six acts of physical abuse (e.g., slapped or thrown something), and 2 acts of sexual abuse (e.g., forced sex). For each of the 18 items, an affirmative response (yes) was coded as perpetrating that specific form of IPV against a current or former partner.

Gender equitable attitudes was assessed using the Gender-Equitable Men (GEM) scale [12]. Participants were asked 8 gender-related statements across five domains gender norms (e.g., “A man should have the final word about decisions in his home”), violence (e.g., “If someone insults me, I will defend my reputation with force if I have to”), sexuality (e.g., “Men need sex more than women do”), toxic masculinity (e.g., “To be a man, you need to be tough”) and reproductive health (e.g., “It’ is a woman’s responsibility to avoid getting pregnant”). Items are listed in **S1 Appendix**. Participants were asked to respond on a 4-point Likert scale ranging from Strongly Agree (1) to Strongly Disagree (4). Responses were summed to create a total score and the total score was categorized into tertiles to represent low, medium, and high levels of gender equitable attitudes and beliefs for the overall sample. A total score for each domain was created as well. The tertiles variable was used in the main analyses and the domain-specific total scores were used in the post-hoc analyses. Creating scores based on tertiles [13, 14] and domains [12, 15] is consistent with previous research.

Men were asked to report socio-demographics including age, education, marital/cohabitating status, employment, and number of children.

### Data analysis

Descriptive statistics (frequencies, means) were conducted to describe characteristics of the analytic sample. All descriptive analyses were conducted using SAS 9.4.

Latent class analyses were based on 18 indicators of lifetime IPV perpetration and were conducted using Mplus 7.0 [16]. Model fit was assessed based on the fit statistics: lowest values for adjusted Bayesian Information Criteria, Lo-Mendell-Rubin likelihood ratio *p*-value < .05, sample size and epidemiologically relevant [17]. An entropy value greater than or equal to 0.80 was considered optimal [18]. Latent classes were named based upon whether the probability of reporting the latent indicator for IPV perpetration was greater than or equal to 0.50. Chi-square tests and ANOVAs were conducted to test whether the demographics significantly differed across latent classes. The prevalence of each latent class by country site was also presented.

Latent class regression was used to test associations between gender equitable attitudes and latent class membership while controlling for age, education, marital/cohabitating status, employment and whether they had children. Odds ratios (ORs), 95% confidence intervals (CI), and p-values <0.05 were used to assess significance in latent class regression models. All analyses accounted for the multistage structure and clustering of sites within countries.

Demographic data was missing for five participants; thus those participants were removed from the final analytic sample (n = 10,173). In the latent regression models, full information maximum likelihood estimation was used to account for missing data.

## Results

### Prevalence of socio-demographics of overall sample

**Table 1** displays characteristics of the overall sample of male participants. The majority of male participants were 34 or younger: 26.3% were 18–24 years and 33.4% were 25–34 years of age and the mean number of children were two. Over two-thirds of the male participants had at least some high school education. The majority were employed (82.8%) and either married or cohabited (66.7%). On average, men had between one and two children across countries.

**Table 1:** Prevalence of demographics, lifetime IPV perpetration and gender equitable attitudes categories among male participants recruited for UN Multi-Country Study on Men and Violence (total sample and by country).

Variables	Total (N = 10,173)	Bangladesh (n = 2,395)	Cambodia (n = 1,812)	China (n = 997)	Indonesia (n = 2,576)	Papua New Guinea (n = 863)	Sri Lanka (n = 1,530)
**Age**							
18–24 years	2674 (26.3)	654 (27.3)	467 (25.8)	127 (12.7)	672 (26.1)	234 (27.1)	520 (33.9)
25–34 years	3392 (33.4)	781 (32.6)	691 (38.1)	295 (29.7)	834 (32.3)	289 (33.5)	506 (33.1)
35–49 years	4103 (40.3)	960 (40.1)	654 (36.1)	575 (57.6)	1070 (41.5)	340 (39.5)	504 (32.9)
**Education**							
No formal education	594 (5.8)	357 (14.9)	171 (9.44)	4 (.4)	18 (.7)	26 (3.0)	18 (1.2)
Primary education	2521 (24.8)	610 (25.5)	750 (41.4)	138 (13.8)	434 (16.9)	464 (53.8)	125 (8.2)
Some high school	3392 (33.3)	728 (30.4)	583 (32.2)	608 (60.9)	570 (22.1)	125 (14.5)	778 (50.8)
Some secondary education	2461 (24.2)	293 (12.2)	126 (6.9)	141 (14.1)	1294 (50.2)	157 (18.2)	450 (29.4)
Post-secondary education	1205 (11.9)	407 (16.9)	182 (10.0)	106 (10.6)	260 (10.1)	91 (10.5)	159 (10.4)
**Employed**	8417 (82.8)	2048 (85.5)	1558 (85.9)	919 (92.2)	2123 (82.4)	632 (73.3)	1137 (74.4)
**Married/Cohabited**	6776 (66.7)	1508 (62.9)	1303 (71.9)	851 (85.4)	1730 (67.2)	553 (64.1)	831 (54.3)
**Children,** *M (SD)*	1.53 (1.85)	1.25 (1.43)	1.77 (1.82)	1.11 (.94)	1.34 (1.55)	2.13 (2.39)	1.97 (2.65)
**Latent Class Indicators of Lifetime IPV Perpetration**							
**Emotional IPV**							
Insulted	2809 (27.6)	403 (25.8)	498 (34.9)	212 (22.3)	1016 (42.0)	513 (70.7)	167 (14.8)
Belittled or Humiliated	1349 (13.3)	340 (21.7)	213 (14.9)	135 (14.2)	252 (10.4)	284 (39.1)	125 (11.0)
Scare or Intimidate Intentionally	2801 (27.5)	475 (30.4)	441 (30.8)	272 (28.6)	875 (36.1)	386 (53.2)	352 (30.9)
Threats of Physical Abuse	1902 (18.7)	472 (30.2)	416 (29.1)	90 (9.5)	228 (9.4)	493 (68.0)	203 (17.8)
Damage Important Things	855 (8.4)	154 (9.8)	96 (6.7)	57 (5.9)	231 (9.5)	208 (28.7)	109 (9.6)
**Economic IPV**							
Prohibited Her Employment	1292 (12.7)	154 (9.9)	365 (25.6)	101 (10.6)	435 (17.9)	134 (18.6)	103 (9.1)
Taken Earning Against Her Will	2401 (23.6)	1240 (79.3)	243 (17.0)	57 (5.9)	487 (20.1)	211 (29.2)	163 (14.3)
Thrown Her Out the House	688 (6.8)	103 (6.6)	111 (7.8)	69 (7.2)	172 (7.1)	175 (24.3)	58 (5.1)
Kept Earnings for Yourself Despite Expenses	1465 (144)	59 (3.8)	434 (30.6)	73 (7.7)	498 (20.7)	310 (42.9)	91 (8.0)
**Physical IPV**							
Slapped or Thrown Something	1818 (17.9)	748 (47.9)	94 (6.6)	285 (29.8)	310 (12.8)	237 (32.7)	144 (12.7)
Pushed or Shoved	1973 (19.4)	596 (38.1)	165 (11.6)	313 (32.8)	340 (14.0)	343 (47.4)	26 (19.1)
Hit with Fist or Something Else	1068 (10.5)	272 (17.4)	90 (6.3)	170 (17.8)	124 (5.1)	335 (46.4)	77 (6.8)
Kicked, Dragged, Beaten, Choken, or Burned	535 (5.3)	123 (7.8)	46 (3.2)	89 (9.3)	55 (2.3)	194 (26.9)	28 (2.5)
Threatened to Use or Actually Used Gun	241 (2.4)	27 (2.7)	28 (1.9)	18 (1.9)	26 (1.1)	117 (16.2)	25 (2.2)
**Sexual IPV**							
Forced Sex	1387 (13.6)	195 (8.2)	156 (8.8)	119 (12.2)	420 (16.6)	357 (41.8)	140 (9.7)
Forced Sex by Marital Status	1577 (15.5)	84 (3.5)	270 (15.2)	146 (15.0)	600 (23.8)	360 (42.3)	117 (8.2)
**Tertiles of Gender Equitable Attitudes**							
Low Tertile	3317 (32.6)	315 (13.2)	1354 (74.7)	40 (4.0)	171 (6.6)	175 (20.3)	1262 (82.5)
Middle Tertile	3668 (36.1)	1332 (55.6)	418 (23.1)	183 (18.3)	1114 (43.2)	372 (43.2)	249 (16.2)
High Tertile	3188 (31.3)	748 (31.2)	40 (2.2)	774 (77.7)	1291 (50.1)	316 (36.6)	19 (1.2)

^a^Proportions (column percentages) are shown, may not equal to 100% due to rounding.

^b^M = mean, SD = standard deviation.

^c^Tertiles of gender equitable attitudes were based on the overall sample.

### Latent class solution

Fit statistics indicate that the five-class model was the optimal solution. Adjusted Bayesian information criterion was small for the five-class model. The entropy of 0.81 for the five-class model also suggested an adequate separation of the classes. The Lo-Mendell-Rubin likelihood ratio test p-value of 0.45 for the five-class model indicated that there were no significant improvements made to the overall fit when a sixth class was included.

**Fig 1:**
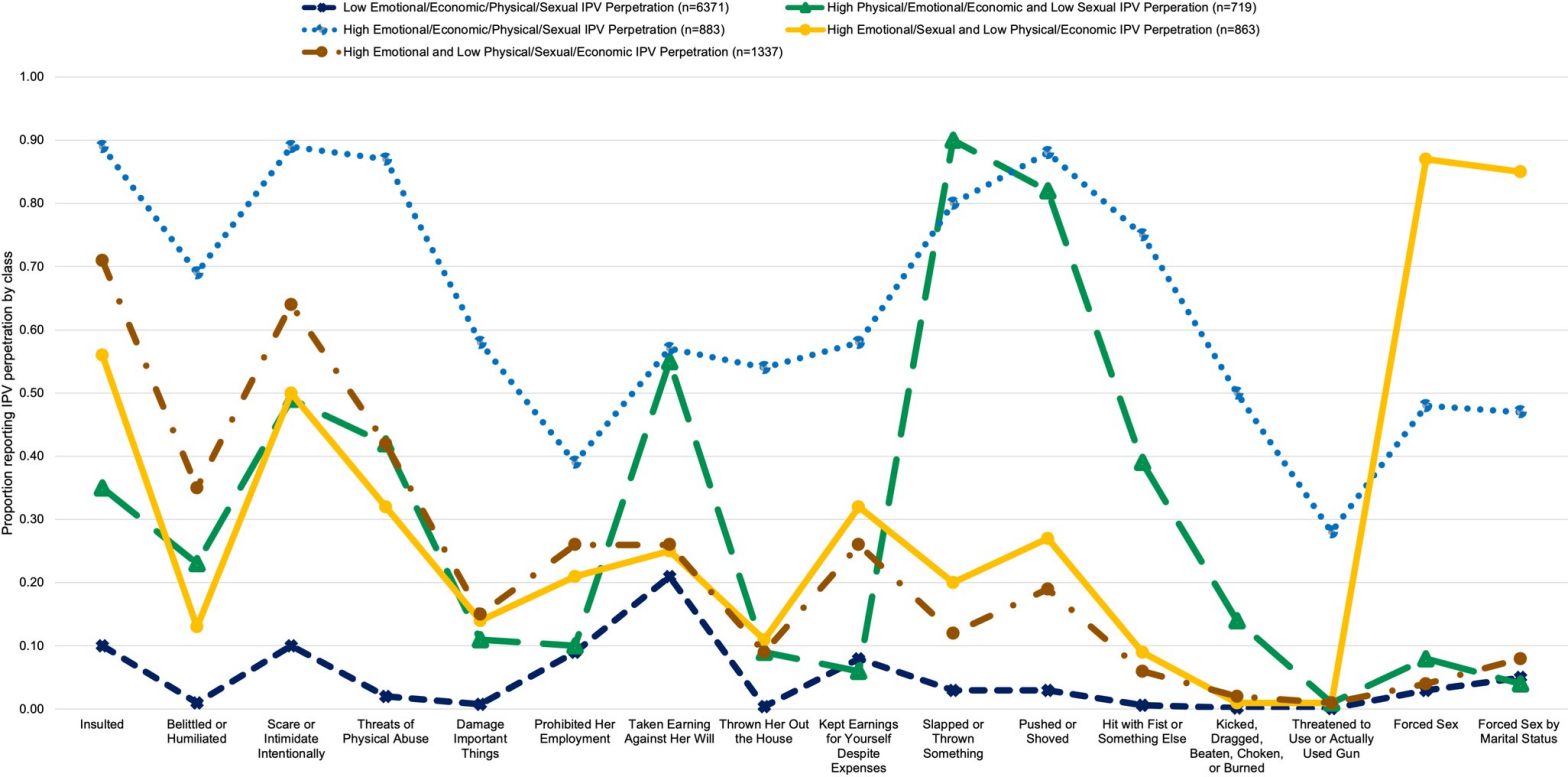
Probability of reporting each IPV perpetration behavior by latent class.

Overall, the five-class model illustrated distinct and meaningful classes. The *Low Physical/Emotional/Economic/Sexual IPV Perpetration* class (hereafter, Low All Forms of IPV Perpetration) represented 62.6% (n = 6371) and was characterized as having a low probability of perpetrating physical, sexual, emotional, and economic IPV (**Fig 1**). The *High Physical/Emotional/Economic and Low Sexual IPV Perpetration* class (hereafter, High Physical/Emotional/Economic IPV Perpetration) represented 7.1% (n = 719) of the sample and was characterized as high probabilities of perpetrating physical, emotional and economic IPV, and low probabilities of perpetrating sexual IPV. The *High Physical/Sexual/Emotional/Economic IPV Perpetration* class (hereafter, High All Forms of IPV Perpetration) represented 8.7% (n = 883) of the sample and was characterized as high probabilities of perpetrating physical, sexual, emotional, and economic IPV. The *High Sexual/Emotional and Low Physical/Economic IPV Perpetration* class (hereafter, High Sexual/Emotional IPV Perpetration) represented 8.5% (n = 863) of the sample and was characterized as high probabilities of perpetrating sexual and emotional IPV, and low probabilities of perpetuating physical and economic IPV. The *High Emotional and Low Physical/Sexual/Economic IPV Perpetration* class (hereafter, High Emotional IPV Perpetration) represented 13.1% (n = 1337) of the sample and was characterized as high probabilities of perpetrating emotional IPV, and low probabilities of perpetrating physical, sexual and economic IPV.

### Socio-demographic characteristics by latent class membership

**Table 2** displays the prevalence of each latent class by country. In all of the countries except Papua New Guinea, at least 60% of the participants in these countries were assigned to the *Low All Forms of IPV Perpetration* class. In Papua New Guinea, the *High Physical/Emotional/Economic IPV Perpetration* class was the most prevalent (32.9%). The *High All Forms of IPV Perpetration* class was the second largest class in Bangladesh (20.9%) and China (18.4%). The *High Emotional IPV Perpetration* class was the second largest class in Cambodia (21.5%), Indonesia (17.8%), and Sri Lanka (8.6%). In Papua New Guinea, the *Low All Forms of IPV Perpetration* class was the second largest class (27.7%).

**Table 2:** Prevalence of latent class membership by country among male participants recruited for UN Multi-Country Study on Men and Violence.

Latent Classes	Total (N = 10,173)	Bangladesh (n = 2,395)	Cambodia (n = 1,812)	China (n = 997)	Indonesia (n = 2,576)	Papua New Guinea (n = 863)	Sri Lanka (n = 1,530)
Low Emotional/Economic/Physical/Sexual IPV Perpetration	6,373 (62.6)	1581 (66.01)	1204 (66.5)	594 (59.5)	1568 (60.9)	239 (27.7)	1187 (77.5)
High Emotional/Economic/Physical/Sexual IPV Perpetration	883 (8.7)	502 (20.9)	28 (1.5)	184 (18.4)	73 (2.8)	30 (3.5)	66 (4.3)
High Physical/Emotional/Economic and Low Sexual IPV Perpetration	721 (7.1)	122 (5.1)	73 (4.0)	59 (5.9)	126 (4.9)	284 (32.9)	57 (3.7)
High Emotional/Sexual and Low Physical/Economic IPV Perpetration	863 (8.4)	47 (1.9)	117 (6.5)	79 (7.9)	350 (13.5)	180 (20.8)	90 (5.8)
High Emotional and Low Physical/Sexual/Economic IPV Perpetration	1,337 (13.1)	143 (10.7)	390 (21.5)	82 (8.2)	459 (17.8)	131 (15.1)	132 (8.6)

**Table 3** displays characteristics of the sample by latent class membership. Latent class membership was significantly associated with sociodemographics and tertiles of gender equitable attitudes. Age was significantly associated class membership (χ^2^  =  581.5, *p* < .0001), of which men aged 35–49 years was the most prevalent across all five latent classes. Education was significantly associated class membership (χ^2^  =  437.9, *p* < .0001), of which men with some high school education were the most prevalent in four of the five latent classes. Employment was significantly associated class membership (χ^2^  =  188.2, *p* < .0001), of which men who were employed were the most prevalent across all five latent classes. Married status was significantly associated class membership (χ^2^  =  873.9, *p* < .0001), of which men who were married were the most prevalent across all five latent classes. Tertiles of gender equitable attitudes was significantly associated class membership (χ^2^  =  113.8, *p* < .0001), of which men in the middle tertile were the most prevalent in three of the five latent classes.

**Table 3:** Prevalence of demographics and gender equitable attitudes by latent class membership among male participants recruited for UN Multi-Country Study on Men and Violence.

Variables	Low Emotional/Economic/Physical/Sexual IPV Perpetration (n = 6,371)	High Emotional/Economic/Physical/Sexual IPV Perpetration (n = 883)	High Physical/Emotional/Economic and Low Sexual IPV Perpetration (n = 719)	High Emotional/Sexual and Low Physical/Economic IPV Perpetration (n = 863)	High Emotional and Low Physical/Sexual/Economic IPV Perpetration (n = 1,337)	*χ *^*2*^ *or F*
**Age**						581.5*
18–24 years	2107 (33.1)	55 (6.2)	100 (13.9)	189 (21.9)	223 (16.7)	
25–34 years	2083 (32.7)	253 (28.6)	260 (36.2)	316 (36.6)	484 (36.2)	
35–49 years	2181 (34.2)	575 (65.1)	359 (49.9)	358 (41.4)	630 (47.1)	
**Education**						437.9*
No formal education	278 (4.3)	137 (15.5)	57 (7.9)	34 (3.9)	88 (6.5)	
Primary education	1384 (21.7)	262 (29.6)	274 (38.1)	227 (26.3)	374 (27.9)	
Some high school	2288 (35.9)	307 (34.77)	179 (24.9)	216 (25.0)	402 (30.1)	
Some secondary education	1579 (24.7)	113 (12.8)	147 (20.4)	291 (33.7)	331 (24.7)	
Post secondary education	842 (13.2)	64 (7.2)	62 (8.6)	95 (11.0)	142 (10.6)	
**Employed**	5059 (79.5)	843 (95.4)	596 (82.9)	738 (85.5)	1181 (88.3)	188.2*
**Married/Cohabited**	3602 (56.5)	839 (95.0)	614 (85.4)	633 (73.3)	1088 (81.4)	873.9*
**Children,** *M (SD)*	1.23 (1.75)	2.08 (1.47)	2.41 (2.08)	1.78 (2.02)	1.99 (1.91)	132.8*
**Tertiles of Gender Equitable Attitudes**						113.8*
Low Tertile	2150 (33.7)	194 (21.9)	223 (30.9)	252 (29.2)	500 (37.4)	
Middle Tertile	2138 (21.9)	416 (47.1)	306 (42.4)	345 (39.9)	464 (34.7)	
High Tertile	2085 (32.7)	273 (30.9)	192 (26.6)	266 (30.8)	373 (27.9)	

*p < .0001

### Associations between gender equitable attitudes and latent class membership

**Fig 2:**
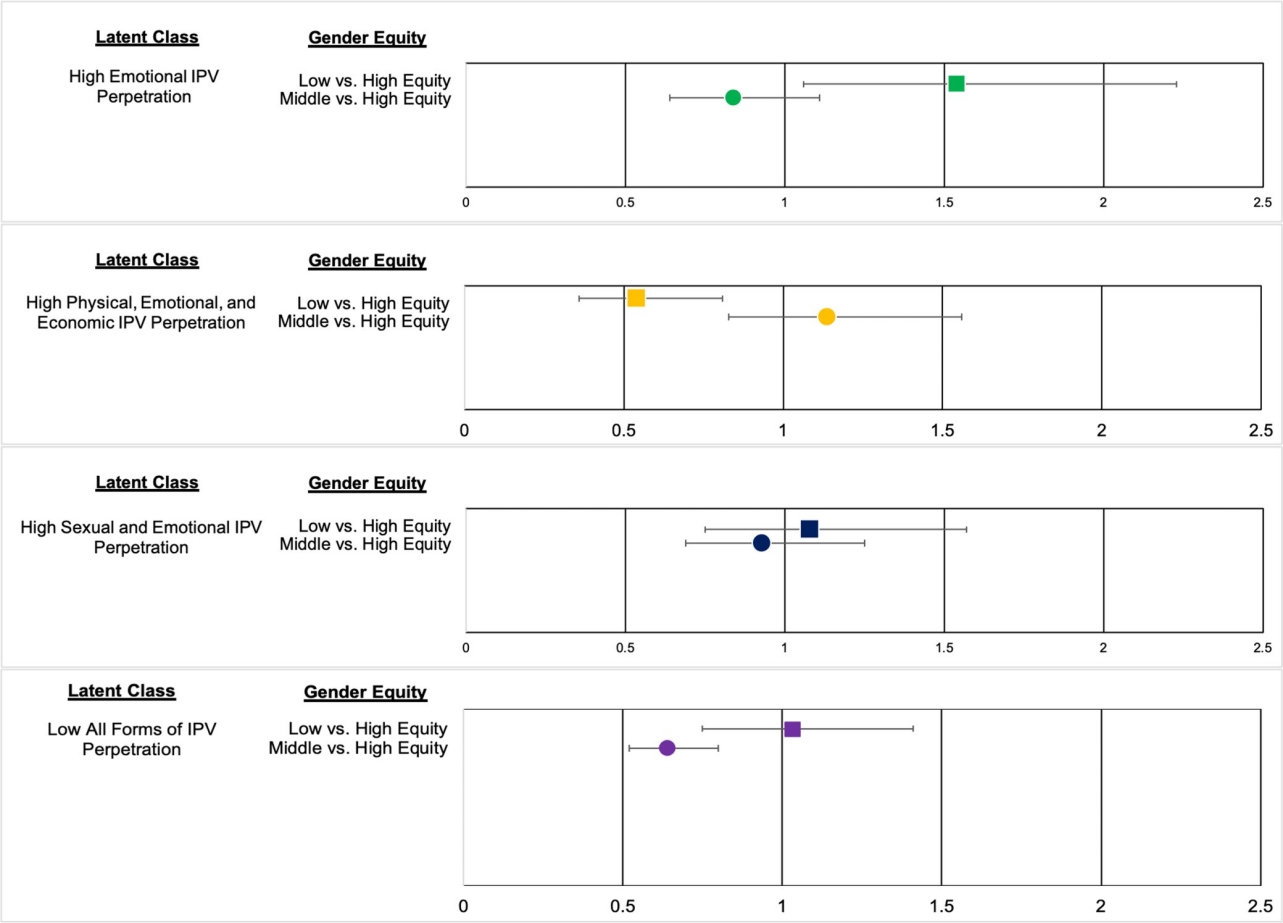
Associations between latent classes and gender equitable attitudes among male participants recruited for UN Multi-Country Study on Men and Violence (total sample). *Note*. High All Forms of IPV Perpetration latent class is not shown because it is the reference group. Covariates include age, education, employment, married/cohabitating, whether they had kids and accounted for multistage clustering.

There were three significant associations between gender equitable attitudes and latent class membership while adjusting for age, education, employment, married/cohabited and number of children (**Fig 2**). In these analyses, men in the *High All Forms of IPV Perpetration* class represented the referent group because our research postulates that men in this group would have more gender inequitable attitudes, and thus all of classes of men who use fewer forms of violence may also have more gender equitable attitudes. The odds of being assigned to the *Low All Forms of IPV Perpetration* class than the *High All Forms of IPV Perpetration* class was lower for men in the middle tertile group than men in the high tertile group for gender equitable attitudes (OR (95% CI) = .64 (.52, .80), *p* = .00). The odds of being assigned to the *High Emotional IPV Perpetration* class than *the High All Forms of IPV Perpetration* class was greater for men in the low tertile group than men in the high tertile group for gender equitable attitudes (OR (95% CI) = 1.54 (1.06, 2.23), *p* = .02). The odds of being assigned to the *High Physical/Emotional/Economic IPV Perpetration* class than the *High All Forms of IPV Perpetration* class was lower for men in the low tertile group than men in the high tertile group for gender equitable attitudes (OR (95% CI) = .54 (.36, .81), *p* = .003).

Post-hoc analyses were conducted to explore how relationships between GEM domains (e.g., toxic masculinity, gender roles, sexuality, violence, and reproductive health) and latent class membership while adjusting for age, education, employment, married/cohabited and number of children (**Table 4**). The odds of being assigned to the *Low All Forms of IPV Perpetration* class than the *High All Forms of IPV Perpetration* class was greater for men with more equitable scores along the toxic masculinity (OR (95% CI) = 1.13 (1.06, 1.25), *p* = .02) and violence domains (OR (95% CI) = 1.69 (1.55, 1.84), *p* = .00); and more inequitable scores along the reproductive health domains (OR (95% CI) = .73 (.67, .79), *p* = .00). The odds of being assigned to the *High Sexual/Emotional IPV Perpetration* class than the *High All Forms of IPV Perpetration* class was greater for men with more equitable scores along the gender norms (OR (95% CI) = 1.66 (1.16, 2.38), *p* = .00) and violence domains (OR (95% CI) = 1.57 (1.39, 1.76), *p* = .00); and more inequitable scores along the reproductive health domain (OR (95% CI) = .83 (.75, .93), *p* = .00). The odds of being assigned to the *High IPV Perpetration* class than the *High All Forms of IPV Perpetration* class was greater for men with more equitable scores along the gender norms (OR (95% CI) = 1.54 (1.11, 2.12), *p* = .00), masculinities (OR (95% CI) = 1.23 (1.10, 1.38), *p* = .00) and violence domains (OR (95% CI) = 1.40 (1.28, 1.54), *p* = .00); and more inequitable scores along the reproductive health domains (OR (95% CI) = .67 (.61, .73), *p* = .00). The odds of being assigned to the *High Physical/Emotional/Economic IPV Perpetration* class than the *High All Forms of IPV Perpetration* class is greater for men with more equitable scores along the sexuality (OR (95% CI) = 1.52 (1.10, 2.08), *p* = .01) and reproductive health domains (OR (95% CI) = 1.32 (1.18, 1.47), *p* = .00).

**Table 4:** Post hoc results of associations between latent classes and domains of gender equitable attitudes among male participants recruited for UN Multi-Country Study on Men and Violence (total sample).

Class Comparisons	OR (95% CI)	*p*
**Low All Forms of IPV Perpetration vs. High All Forms of IPV Perpetration**		
Gender	1.23 (.95, 1.60)	.11
Sexuality	1.01 (.79, 1.28)	.94
Masculinity	1.13 (1.06, 1.25)	.02
Reproductive Health	.73 (.67, .79)	.00
Violence	1.69 (1.55, 1.84)	.00
**High Sexual/Emotional IPV Perpetration vs. High All Forms of IPV Perpetration**		
Gender	1.66 (1.16, 2.38)	< .01
Sexuality	1.05 (.77, 1.44)	.76
Masculinity	.94 (.83, 1.08)	.43
Reproductive Health	.83 (.75, .93)	< .01
Violence	1.57 (1.39, 1.76)	.00
**High Emotional IPV Perpetration vs. High All Forms of IPV Perpetration **		
Gender	1.54 (1.11, 2.12)	< .01
Sexuality	1.07 (.79, 1.44)	.66
Masculinity	1.23 (1.10, 1.38)	.00
Reproductive Health	.67 (.61, .73)	.00
Violence	1.40 (1.28, 1.54)	.00
**High Physical/Emotional/Economic IPV Perpetration vs. High All Forms of IPV Perpetration**		
Gender	.91 (.64, 1.31)	.62
Sexuality	1.52 (1.10, 2.08)	.01
Masculinity	.97 (.83, 1.13)	.72
Reproductive Health	1.32 (1.18, 1.47)	.00
Violence	.97 (.83, 1.13)	.70

^a^Covariates include age, education, employment, married/cohabitating, whether they had kids and accounted for multistage clustering.

^b^Low All Forms of IPV Perpetration (n = 6,371); High Sexual/Emotional IPV Perpetration (n = 863); High Emotional IPV Perpetration (n = 1,337); High Physical/Emotional/Economic IPV Perpetration (n = 719); and High All Forms of IPV Perpetration (n = 883).

## Discussion

To date, empirical IPV research has largely ignored the presence of a potential pattern or lack thereof in men’s IPV perpetration behaviors. This is one of the first studies to examine distinct patterns of IPV perpetration among men in the LMIC context as well as assess gender attitudes and norms as a correlate of those distinct patterns. Using latent class analysis, the present findings indicate five distinct patterns of IPV perpetration among men in the Asia-Pacific region. More than one in three men were characterized in a class indicative of some form of IPV perpetration. Men’s use of IPV has been extensively examined in LMICs [5, 6, 19], however, our study adds to this robust literature by utilizing latent class analysis to identify distinct patterns in the Asia-Pacific region. Further, our study showed significant associations between men’s endorsement of gender equitable attitudes and patterns of IPV perpetration. Our findings suggest that men’s views on gender equity could influence the heterogeneity in men’s use of IPV perpetration. Moreover, distinct domains of gender inequity (e.g., sexuality, toxic masculinity) could provide additional context for some of these associations. Collectively, these findings illustrate ways in which gender inequities contribute to distinct IPV perpetration behaviors exhibited by men in the Asia-Pacific region. Gender-transformative programming and interventions may improve reductions in IPV perpetration among men in the Asia-Pacific region by tailoring intervention components to men’s distinct pattern of IPV perpetration.

Our findings illustrate significant heterogeneity in men’s perpetration of IPV as five classes emerged. For instance, among the four latent classes with a high probability of IPV perpetration, emotional IPV perpetration (e.g., belittling, insults) was the only form of IPV that was present across four latent classes. This finding suggests that emotional IPV is a prevalent form of violence perpetrated by men in the Asia-Pacific region. Emotional IPV also tended to co-occur with other forms of IPV (e.g., physical, sexual), but it is worth noting that one latent class was characterized as men who only used emotional IPV against women (i.e., *High Emotional IPV Perpetration*). Emotional abuse was prevalent among IPV perpetrators, but there was variation in physical, sexual, and economic IPV perpetrated by men. For example, high levels of physical IPV and high levels of economic IPV tended to co-occur and this was present in two classes (i.e., *High Physical/Emotional/Economic IPV Perpetration*; and *High All Forms of IPV Perpetration*). Moreover, there was a latent class characterized as high levels of sexual and emotional IPV perpetration, but low levels of physical and economic IPV perpetration (i.e., *High Emotional/Sexual IPV Perpetration*). In LMICs, the prevalence of sexual IPV reported by ever-partnered women ranges between 6–59% [20]. This, in combination with our findings, suggests that men who perpetrate sexual IPV are likely to also use emotional abuse towards their intimate partners. Lastly, the largest latent class was characterized as men who reported little to no IPV perpetration against female intimate partners (i.e., *Low All Forms of IPV Perpetration*). Although the majority of men in this sample were classified in the nonviolent latent class, nearly 37% of men were classified in an IPV perpetration class which is consistent with global estimates of IPV victimization experienced by women [20].

It is worth noting that some classes of men’s IPV perpetration are consistent with previous research identifying classes of women’s IPV victimization in LMICs. For example, a study in Uganda found four distinct classes of women based on their IPV experiences which include a low violence class, high physical violence class, high sexual violence class, and a severe sexual and physical violence class [21]. Although the aforementioned study focused on physical and sexual IPV [21], a general pattern of IPV behaviors can be identified. In particular, in our study based on men’s perpetration and the Ugandan study based on women’s victimization, there were classes characterized as low perpetration or victimization; high perpetration or victimization; in addition to distinct classes based on combinations of IPV subtypes.

There were significant associations between men’s endorsement of gender equitable attitudes and their latent class membership. Generally, our findings are consistent with previous literature on gender equitable attitudes [12, 22, 23], suggesting that more gender equitable attitudes relate to less violence being perpetrated by men. For example, men in the low and middle tertiles for gender equitable attitudes had a greater likelihood of being classified in the *High All Forms of IPV Perpetration* class than the *High Physical/Emotional/Economic IPV Perpetration* and *Low All Forms of IPV Perpetration* classes, respectively. These findings suggest that gender equitable attitudes are important contributors of men’s typologies of IPV perpetration. Socially constructed roles between women and men can manifest as power differentials and result in men’s perpetration of violence against women [22]. Gender-transformative interventions that address how social constructions of masculinity and femininity shape and define gender roles in relationships may help reduce men’s likelihoods of using all types of IPV aging women [24, 25].

It is noteworthy that men in the low tertile for gender equitable attitudes had a greater odds of being classified in the *High Emotional IPV Perpetration* class than the *High All Forms of IPV Perpetration* class. It is possible that this unique finding could be explained by previous research examining how different risk and protective factors are associated with different types of IPV perpetration. For example, a previous study found that men who strongly endorsed sexual cultural scripting norms (e.g., socially constructed sexual roles and expectations between women and men) had a greater likelihood of perpetrating emotional IPV; while men who strongly endorsed traditional masculine norms (e.g., societal views of men’s actions, feelings, behaviors) had a greater likelihood of perpetrating physical IPV [26]. Building from this previous research, it is possible that men with strong endorsement of sexual cultural scripting norms but weaker endorsement of traditional masculine norms may perpetrate emotional IPV (e.g., belittling) towards their female partner, if she resists these cultural scripting norms (e.g., rejects his sexual advances). It is also possible that men who would be classified in the *High Emotional IPV Perpetration* class have differential access to protective factors (i.e., more social support) that prevent perpetration of physical, sexual, and economic IPV than men in the *High All Forms of IPV Perpetration* class. It would be useful for future research to examine differences in protective factors based upon men’s typologies of IPV perpetration in order to inform strengths-based adaptive primary prevention interventions.

Notably, our post-hoc analyses indicated that men with equitable gender attitudes across all domains (i.e., toxic masculinity, gender roles, reproductive health, violence and sexuality) tended to be classified in a typology with fewer types of IPV perpetrated but there were some key differences. Specifically, every domain was not significantly associated with men’s typologies. For example, equitable attitudes in the domains of violence, masculinity and gender did not significantly correlate with men in the *High Physical/Emotional/Economic IPV Perpetration* class vs. the *High All Forms of IPV Perpetration* class. Further, men with more equitable attitudes in the sexuality and reproductive health domains tended to be classified in the *High Physical/Emotional/Economic IPV Perpetration* class than the *High All Forms of IPV Perpetration* class. It is possible that specific domains of gender inequitable attitudes are differentially impacting men’s motivations to perpetrate each IPV type. Additional research is needed to assess which mechanisms, if any, may explicate the differential impacts of gender equitable attitude domains on men’s typology of IPV perpetration. It may also be useful for intervention scientists to examine men’s typology of IPV perpetration as an effect modifier of intervention efficacy. If significant, it may have important considerations for gender transformative interventions. Gender transformative interventions seek to reshape gender relations and address social and structural drivers of gender inequality [24]. For example, gender transformative interventions could promote gender equitable attitudes through coach-led training sessions for young male athletes [27]. In relation to the study’s findings, gender transformative interventions may need to tailor the dose and type of intervention components (e.g., gender equitable domains) to men’s typologies of IPV perpetration, such that men in the *High Physical/Emotional/Economic IPV Perpetration* class receive a lower intervention dose in regards to equitable attitudes in the sexuality and reproductive health than men in the *High All Forms of IPV Perpetration* class. These findings also suggest the importance of investigating differential treatment effects by latent class as part of the rapidly growing body of work on IPV prevention in LMICs.

The present study has some limitations. This study is based on the collection of self-reported data, and IPV perpetration may be under-reported, if perceived as a private matter. Under-reporting of IPV perpetration may result in misclassification of latent class membership among this sample, which could result in a lower prevalence across the latent classes and weaken our regression estimates. The study was first conducted in Bangladesh and as a result, the sexual IPV questions were expanded to include coerced sex; therefore, the sexual IPV questions in Bangladesh were different from the questions asked in the other countries, which may have influenced the prevalence. The cross-sectional nature of the data makes it difficult to assess causality. In particular, identifying the directionality of the relationship between gender inequitable attitudes and IPV perpetration is difficult because both of these constructs could reinforce one another (e.g., IPV perpetration could result in stronger adherence to gender inequitable attitudes; but also strong acceptance of gender inequitable attitudes could increase the likelihood of IPV perpetration). Although we accounted for the clustering effects of the sampling method, this study did not examine these associations by country.

## Conclusions

Despite these limitations, to our knowledge, this is the first study to use latent class analysis to identify men’s typology of intimate partner violence perpetration and examine its association with gender equitable attitudes. Our findings suggest that IPV perpetration is prevalent among men in the Asia-Pacific region, and five latent classes were identified based on men’s perpetration of IPV. In general, men with more gender equitable attitudes tended to be in a latent class with fewer types of IPV perpetrated than men with less gender equitable attitudes. There is also evidence to suggest that specific domains of gender equitable attitudes may differentially impact men’s typology of IPV perpetration. Findings from this study highlight the importance of continual support for gender transformative interventions addressing IPV perpetration, however an adaptive approach that is personalized to men’s typology of IPV perpetration may have greater impact to reduce violence and promote safety for women in the Asia-Pacific region.

## Supporting information

S1 Appendix (DOCX)
