# Supplementary material for: Latent classes of men’s intimate partner violence perpetration and attitudes towards gender norms: A UN multi-country, cross-sectional study in Asia and the Pacific
Source: PLoS One. 2022 Sep 26;17(9):e0264156. doi: 10.1371/journal.pone.0264156 (PMC9512213; doi:10.1371/journal.pone.0264156)
Supplement: S1 Appendix — (DOCX) [file pone.0264156.s001.docx]

**Appendix A**

GEM Scale

- A woman’s most important role is to take care of her home and cook for her family.
- Men need sex more than women do.
- There are times when a woman deserves to be beaten.
- It is a woman’s responsibility to avoid getting pregnant.
- A woman should tolerate violence in order to keep her family together.
- You would be outraged if your wife asked you to use a condom.
- If someone insults you, you will defend your reputation, with force if you have to.
- To be a man, you need to be tough
